# Human Adenovirus Type 55 Distribution, Regional Persistence, and Genetic Variability

**DOI:** 10.3201/eid2607.191707

**Published:** 2020-07

**Authors:** Jun Hang, Adriana E. Kajon, Paul C. F. Graf, Irina Maljkovic Berry, Yu Yang, Mark A. Sanborn, Christian K. Fung, Anima Adhikari, Melinda S. Balansay-Ames, Christopher A. Myers, Leonard N. Binn, Richard G. Jarman, Robert A. Kuschner, Natalie D. Collins

**Affiliations:** Walter Reed Army Institute of Research, Silver Spring, Maryland, USA (J. Hang, I.M. Berry, Y. Yang. M.A. Sanborn, C.K. Fung, A. Adhikari, L.N. Binn, R.G. Jarman, R.A. Kuschner, N.D. Collins);; Lovelace Respiratory Research Institute, Albuquerque, New Mexico, USA (A.E. Kajon);; US Naval Health Research Center, San Diego, California, USA (P.C.F. Graf, M.S. Balansay-Ames, C.A. Myers)

**Keywords:** adenovirus, acute respiratory disease, emerging infectious disease, genetic variation, whole genome sequencing, viruses, United States

## Abstract

Human adenovirus type 55 (HAdV-55) causes acute respiratory disease of variable severity and has become an emergent threat in both civilian and military populations. HAdV-55 infection is endemic to China and South Korea, but data from other regions and time periods are needed for comprehensive assessment of HAdV-55 prevalence from a global perspective. In this study, we subjected HAdV-55 isolates from various countries collected during 1969–2018 to whole-genome sequencing, genomic and proteomic comparison, and phylogenetic analyses. The results show worldwide distribution of HAdV-55; recent strains share a high degree of genomic homogeneity. Distinct strains circulated regionally for several years, suggesting persistent local transmission. Several cases of sporadic introduction of certain strains to other countries were documented. Among the identified amino acid mutations distinguishing HAdV-55 strains, some have potential impact on essential viral functions and may affect infectivity and transmission.

Acute respiratory diseases (ARD) are caused by numerous viral pathogens, including several human adenovirus (HAdV) types. Respiratory HAdV infections range from mild to severe and are fatal in some cases. HAdV-associated respiratory disease has threatened military readiness, and an increasing number of outbreaks and isolated cases documented in civilian communities in the United States and other countries (*1*–*4*) indicate that it is an emerging threat to public health. The live oral HAdV type 4 and type 7 vaccines are highly effective against the 2 dominant types causing ARD outbreaks in the military environment, but they elicit type-specific immunity and are restricted to use in the US military (*5*–*7*). ARD caused by other HAdV types, such as HAdV types 3, 14, and 21, occurs sporadically in military basic training facilities (*8*–*10*). An increasing number of new HAdV types have been described, indicating the potential emergence of new HAdV-associated diseases (*11*–*13*).

HAdV-55 was initially identified in respiratory isolates originating in China during 1965–1981 as serotype 11a, a distinct genomic variant of serotype 11, by its distinct *Bam*HI digestion profile compared with the HAdV-11 prototype strain Slobitski (*14*). This unique subspecies HAdV-B2 genotype was designated HAdV-55 (P14H11F14) in 2013 after the bioinformatics analysis of complete genome sequences revealed that the genome consists of a HAdV-14 backbone with a portion of the hexon gene from HAdV-11 (*15*–*17*). HAdV-55 has reemerged as a prevalent ARD pathogen, with endemic circulation reported in China and South Korea since 2006 (*18*–*21*). HAdV-55 whole-genome sequences (WGS) available in GenBank are mainly representative of strains from China and South Korea, but also include strains from Argentina, Egypt, and Singapore (Table 1) (*22*–*25*). More data for HAdV-55 isolates from other global locations and years are needed to enable a comprehensive investigation of the spectrum of intratypic genetic variability, phylogeny, and evolution of HAdV-55, and to apply the knowledge to epidemiology and preventive measures. We acquired WGSs of 72 HAdV-55 clinical isolates from 1969–2018 in 6 countries, and conducted genomic, proteomic, and phylogenetic analyses to reveal unique characteristics of HAdV-55 and identify amino acid residue differences between strains.

**Table 1:** HAdV type 55 strains from other studies used in genomic analysis for study of virus distribution, regional persistence, and genetic variability

HAdV-55 strain	Collection year	Collection date	Country	GenBank accession no.
Human/EGY/ak37_AdV11a/2001/55[P14H11F14]	2001	May 1	Egypt	JX423385
Human/ARG/ak36_AdV11a/2005/55[P14H11F14]	2005	Jan 1	Argentina	JX423384
SGN1222	2005	May 26	Singapore	FJ597732
QS-DLL	2006	Apr	China	FJ643676
CQ-814	2010	Aug 18	China	JX123027
HAdV-B/CHN/BJ01/2011/55[P14H11F14]	2011	Mar 24	China	JX491639
Shanxi/QZ01/2011		Dec 5	China	KJ883522
CQ-1657		Apr 22	China	JX123028
Shanxi-Y16		UNK	China	MK123979
Human/CHN/AQ-1/2012/55[P14/H11/F14]	2012	Apr 18	China	KP279748
HAdV-B55 XZ2012-492		Apr 26	China	KC857701
CQ-2903		Jan 8	China	JX123029
Hebei/BD01/2012		Feb 11	China	KP896478
Hebei/BD6728/2013	2013	Apr 6	China	KJ883520
TJ-2013-90		Jan 14	China	KF908851
TY12		UNK	China	MK123980
TY26		UNK	China	MK123981
Liaoning/LS01/2013		Feb 25	China	KP896483
Tianjin/TJ01/2013		Jan 15	China	KP896484
Hebei/BD6729/2013		Apr 7	China	KJ883521
JS201501	2015	Nov 12	China	KX289874
100-GD_CHN_2016	2016	Jun	China	KY780931
73-GD_CHN_2016		Jun	China	KY780933
60-GD-2016		Jun 30	China	KY070248
Yunnan/KM04/2016		Jun 8	China	KY002685
AFMC 16-0011	2016	Feb 23	South Korea	KX494979
267	2018	Jun	China (Guangzhou)	MK123978

*HAdV, human adenovirus; UNK, unknown.

## Materials and Methods

### HAdV-55 Strains, DNA Extraction, Genome Sequencing

The HAdV-55 isolates included in this study were acquired from 2 main sources: the archival collection of isolates at Lovelace Respiratory Research Institute (LRRI), Albuquerque, NM, USA, gathered through collaborative surveillance efforts funded by the US Department of Defense’s Global Emerging Infections Surveillance and Response System; and the HAdV-positive specimen collection of the Naval Health Research Center—Operational Infectious Disease (NHRC-OID, San Diego, CA, USA), conducts surveillance of febrile respiratory illness among military personnel and their dependents in the Pacific Rim at Commander US Fleet Activities Yokosuka, Yokosuka, Japan (Table 2). At LRRI, we performed viral isolation in A549 cell cultures, purification of HAdV genomic DNA, and molecular typing by restriction enzyme analysis or by PCR and Sanger sequencing of hexon and fiber genes as previously described (*8*,*16*). At Walter Reed Army Institute of Research (Silver Spring, MD, USA), viral DNA samples received from LRRI were subjected to next-generation sequencing (NGS) fragment library preparation using QIAseq FX DNA Library Kit (QIAGEN, https://www.qiagen.com), followed by sequencing by using MiSeq Reagent Kit version 3 (600-cycle) and MiSeq sequencer (Illumina, https://www.illumina.com) (*26*). At the Pacific Rim Surveillance Hub (PRSH) of NHRC-OID, respiratory samples collected from persons meeting case definition for febrile respiratory illness were tested on the FilmArray Respiratory Pathogen Panel (Biofire Diagnostics, https://www.biofiredx.com), a multiplex panel consisting of 21 respiratory viral and bacterial pathogens. Adenovirus-positive samples were submitted to OID from Brian Allgood Army Community Hospital (BAACH), Seoul, South Korea. All samples from PRSH and BAACH sent to OID underwent further characterization that included additional typing by PCR amplification and sequencing of hypervariable region 7 of the hexon gene HVR7 as previously described (*27*). Clinical samples that failed to sequence were reflex-tested on type-specific assays that targeted the hexon gene, and were also inoculated in A549 cells to attempt viral isolation. We subjected all confirmed HAdV isolates to whole-genome NGS using Illumina Nextera XT library preparation kit and MiSeq System.

**Table 2:** HAdV type 55 strains used for genomic analysis for study of virus distribution, regional persistence, and genetic variability*

Strain	Collection year	Collection site	Country	Source	GenBank accession no.
HAdV-11/14 strain 273	1969	Military camp	Spain	LRRI§	MN654395
CADOH VRDL 76–0669	1976	California	USA	LRRI	MN654394
CDC 97026382	1997	South Dakota	USA	LRRI	MN654392
NAMRU3-E3	2000	Alexandria	Egypt	LRRI	MN654380
NAMRU3-E4	2000	Alexandria	Egypt	LRRI	MN654381
NAMRU3-E6	2000	Alexandria	Egypt	LRRI	MN654385
NAMRU3-E66	2002	Alexandria	Egypt	LRRI	MN654382
NAMRU3-E72	2000	Alexandria	Egypt	LRRI	MN654383
NAMRU3 2005–909685	2005	Cairo	Egypt	LRRI	MN654390
NAMRU3 2005–908017	2005	Cairo	Egypt	LRRI	MN654391
NAMRU3 2007–905716	2007	Cairo	Egypt	LRRI	MN654386
NAMRU3 2008–905223	2008	Cairo	Egypt	LRRI	MN654384
NAMRU3 2009–908968	2009	Cairo	Egypt	LRRI	MN654387
SNG1218	2005	Singapore	Singapore	LRRI	MN654388
SNG1223	2005	Singapore	Singapore	LRRI	MN654389
WPAFB24	2009	BAACH†	South Korea	LRRI	MN654379
WPAFB25	2009	BAACH	South Korea	LRRI	MN654378
WPAFB48	2009	BAACH	South Korea	LRRI	MN654377
WPAFB69	2009	BAACH	South Korea	LRRI	MN654375
WPAFB75	2009	BAACH	South Korea	LRRI	MN654376
WPAFB415	2012	Misawa AB‡	Japan	LRRI	MN654393
NHRC557006	2017	CFA Yokosuka¶	Japan	NHRC	NA#
NHRC isolates, n = 50	2017, 2018	BAACH	South Korea	NHRC	NA#

*An additional 27 full genome sequences from GenBank included in the analysis are listed in Table 1. HAdV, human adenovirus. †Brian Allgood Army Community Hospital at US Army Garrison Yongsan, Seoul, South Korea. ‡Misawa Air Base, Misawa, Aomori, Japan. §LRRI archival collection of isolates generated through collaborations with NHRC, United States Air Force School of Aerospace Medicine, US Naval Medical Research Unit No. 3, US Centers for Disease Control and Prevention, and California Department of Health. ¶Commander US Fleet Activities Yokosuka, Yokosuka, Japan. #Isolates were fully genome sequenced and found to be identical to the South Korea HAdV-55 strains.

### Whole-Genome Sequence Assembly, Annotation, and Comparison

We first analyzed NGS data acquired at Walter Reed Army Institute of Research with an in-house de novo pathogen discovery pipeline to identify possible mix of viruses (*28*). We assembled full genome sequences using NGS_Mapper (https://github.com/VDBWRAIR/**ngs_mapper**) an in-house reference mapping pipeline built on BWA-MEM assembler (H. Li, unpub. data, https://arxiv.org/abs/1303.3997v2). We used Geneious R10 (Biomatters Ltd., https://www.geneious.com) and Integrative Genomics Viewer (IGV) software (Broad Institute, https://igv.org) for manual curation of the assembly, sequence alignment, annotation of genes, etc. We used the complete genome sequence of HAdV-55 strain QS-DLL/China/2006 (GenBank accession no. FJ643676) (*29*,*30*) as reference in reference mapping, annotation, and sequence comparison. We analyzed NGS data acquired at NHRC using the EDGE (Empowering the Development of Genomics Expertise) Bioinformatics Platform (https://edge.readthedocs.io) (*31*) and Lasergene software suite (DNASTAR, Inc., https://www.dnastar.com), and performed sequence alignment using BLAST (http://blast.ncbi.nlm.nih.gov/Blast.cgi).

### Phylogenetic and Proteomic Analysis

We used MAFFT and MEGA7 (*32*) to align HAdV-55 genomic sequences. We determined general time reversible gamma-distributed invariant models of evolution using jModelTest2 (https://github.com/ddarriba/**jmodeltest2)**, and inferred a maximum likelihood phylogenetic tree using PhyML in MEGA7 (http://www.megasoftware.net), with subtree pruning and regrafting and nearest-neighbor interchange tree search and Shimodiara-Hasegawa approximate likelihood ratio test for node confidence values.

We input the nucleotide sequence alignment and the GenBank feature table for the reference strain QS-DLL/China/2006 (FJ643676) into an in-house pipeline that annotates the sequences. To aid in visualization and comparison, we concatenated and aligned the annotated protein sequences using MUSCLE (http://www.drive5.com/muscle) in Geneious R10. We removed redundant identical sequences for further analysis. We visualized mismatches with an augmented version of the output using Highlighter software (https://www.hiv.lanl.gov/content/sequence/HIGHLIGHT/highlighter_top.html) (*33*).

## Results

HAdV55 has been detected worldwide for decades (Table 1). We obtained complete genome sequences for a set of diverse HAdV-55 strains originating in 6 countries over many years and deposited them in GenBank under accession nos. MN654375–MN654395 (Table 2). The characterized strains include strain 273/Spain/1969, originally identified as an intermediate variant 11/14 and isolated during an outbreak of ARD in the Spanish military (*34*); it is the earliest available HAdV-55 isolate and is therefore considered the prototype strain. In addition, the examined collection includes 2 strains isolated from civilians in the United States, 76-0669/USA/CA/1976 and 97026382/USA/SD/1997, the strain isolated during a large ARD outbreak in a civilian job training facility in South Dakota (*35*); 10 HAdV-55 strains from 2 major cities in Egypt; 2 strains from a Singapore military base; 2 HAdV-55 strains isolated in different locations and years in Japan; and 50 recent strains from South Korea.

**Figure 1:**
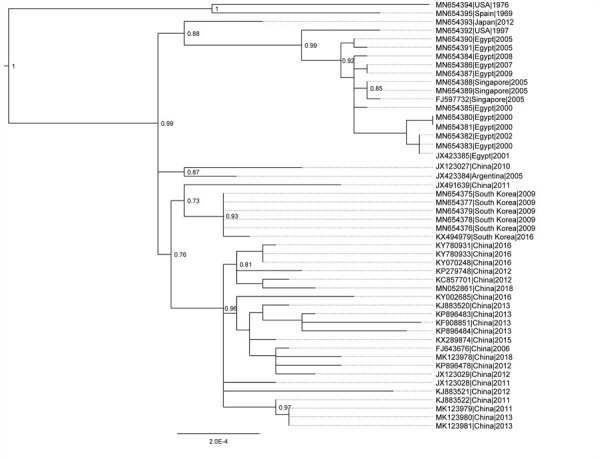
Phylogeny of HAdV-55 based on whole-genome sequences for study of virus distribution, regional persistence, and genetic variability. The phylogenetic tree was generated using the maximum-likelihood method with subtree pruning and regrafting and nearest-neighbor interchange tree search and the Shimodiara-Hasegawa approximate likelihood ratio test for node confidence values. Node confidence values were estimated using approximate likelihood ratio test and the tree was rooted on a HAdV-14 clade as an outgroup (not shown). GenBank accession numbers for isolates are provided. Scale bar indicates node confidence value. HAdV, human adenovirus.

The genome sequences of HAdV-55 strains are highly similar, with 132 or fewer nucleotide differences out of the 34.8 kb genome (i.e., genomic nucleotide divergence <0.38% among all the strains). The inferred phylogenetic tree of HAdV-55 lineage shows the examined strains mostly clustered together by collection country (South Korea, China, Singapore, and Egypt), rather than collection year (Figure 1). The results demonstrate the long-term regional persistence of HAdV-55 infection, which appears to span for years. The 3 isolates collected in 2005 from Singapore were identical to each other and located within the clade of Egypt isolates from 2000–2009. We detected <9 nt differences among all 14 Egypt and Singapore isolates examined (12 from this study; 2 from GenBank). The genome sequences of isolate NHRC557006/Japan/2017 and all the South Korea isolates from this study and in GenBank were found to be identical, except for the differences in the length of the noncoding poly(A) or poly(T) sequences. A previous isolate, WPAFB415/Japan/2012, was phylogenetically distinct from isolate NHRC557006/Japan/2017 and South Korea strains, with proximity with the Egypt strains. Likewise, isolates from South Korea clustered in a monophyletic clade located proximate to the clade of China strains. Another isolate from China was found outside of the Chinese clade and was more related to a strain from Argentina. Nevertheless, we show a highly localized persistence of HAdV-55. GenBank had 1 available genome sequence for an HAdV-55 strain from Argentina, ak36_AdV11a/ARG/2005 (accession no. JX423384); however, Kajon et al. (*16*) performed thorough restriction enzyme analysis characterization and compared hexon and fiber gene sequences for 7 Argentina HAdV-55 isolates collected in 2000–2005. Their results suggest these are isolates of a single strain. Of interest, we found the virtual restriction profiles with *BamH*I, *Bcl*I, *Bgl*II, *Bst*EII, *Hind*III, *Hpa*I, *Pst*I, *Sma*I, and *Xba*I derived from genome sequence JX423384 to be 100% identical to the patterns reported for HAdV-55 strains by Kajon et al., suggesting a single strain was responsible for multiple ARD cases in Argentina.

**Figure 2:**
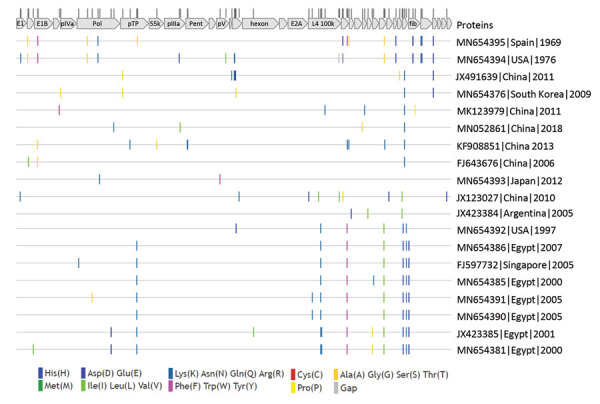
Protein sequence variations among human adenovirus type 55 strains for study of virus distribution, regional persistence, and genetic variability. Protein sequences were concatenated and aligned. Amino acid differences compared with the consensus were visualized with Highlighter software (https://www.hiv.lanl.gov/content/sequence/HIGHLIGHT/highlighter_top.html). Redundant sequences are not shown. The locations of proteins and variant amino acid residues are shown at top. GenBank accession numbers for isolates are provided. Ala, alanine; Arg, arginine; Asn, asparagine; Asp, aspartate; Cys, cysteine; Gln, glutamine; Glu, glutamate; Gly, glycine; His, histidine; Ile, isoleucine; Leu, leucine; Lys, lysine; Met, methionine; Phe, phenylalanine, Pro, proline; Ser, serine; Thr, threonine; Trp, tryptophan; Tyr, tyrosine; Val, valine.

We compared amino acid sequences of all open reading frames (ORF) among HAdV-55 strains (Figure 2; Appendix Table) and found very few amino acid differences. Out of 10,925 aa in the annotated 37 proteins, only 2–15 aa residues in each strain are different from consensus protein sequences (amino acid variation *<*0.14%) among the HAdV-55 strains. No single annotated protein had >2 amino acid differences compared with the consensus except BJ01/CHN/2011 (GenBank accession no. JX491639), which had 6 amino acid substitutions in the 246 aa L3 pVI 26.6 kDa protein and 3 amino acid mutations in the 812 aa L4 100K protein (Appendix Table). Seven proteins, L2 pVII 21.3 kDa protein, L3 pVI 26.6 kDa protein, L3 23K 23.7 kDa protein, E2A 58.3 kDa DNA binding protein, L4 pVIII 25 kDa protein, E4 ORF3 13.6 kDa protein, and E4 ORF2 14.3 kDa protein, were identical among all the examined strains. 

Our analyses showed that hexon, fiber, and penton base proteins do not have more amino acid residue changes when compared with other proteins (Figure 2). We detected a few insertions and deletions in intergenic untranslated regions in several genomes. The strain USA/1976 has 2 deletions (of 3 aa each) in the coding sequences for L4 100K/91 kDa protein and L4 22K/21.6 kDa protein (Appendix Table). It is remarkable that there were no amino acid differences among South Korea isolates from 2009–2018. Despite the very few amino acid substitutions, most residue changes resulted in amino acids of different chemical structures, which may potentially affect protein functionality (Appendix Table). Several amino acid mutations were strain-specific and not seen in other strains or other related HAdV types. One specific example is that the P18S mutation in the terminal protein precursor (pTP) was only found in the South Korea strain. This position was highly conserved (a proline) in all other examined adenoviruses including HAdVs of species B and related simian or gorilla adenoviruses (GenBank accession nos. AP_000267, AP_000305, YP_006272955, ADQ38372). The strain BJ01/CHN/2011 was phylogenetically more closely related to the South Korea strain and had the same pTP sequence with the P18S mutation.

We obtained a total of 51 HAdV-55 isolates, 50 recovered from US military active duty personnel in South Korea and 1 from a US military dependent in Japan, through PRSH efforts. Whole-genome sequencing and sequence data analysis confirmed all strains to be identical to the South Korea strain in full agreement with the recent reports on HAdV-55 circulation in South Korea and the high number of ARD cases documented among the South Korea military (*18*,*36*).

## Discussion

Recent reports describing large numbers of HAdV-55-associated ARD cases in both China and South Korea, including outbreaks and some deaths in both civilian and military communities, have raised concerns about the possibility of global transmission events, similar to those described for severe acute respiratory syndrome or Middle East respiratory syndrome (*21*,*37*–*43*). Outbreaks and isolated cases of HAdV-55-associated ARD have been reported in the literature in other countries, such as Turkey (*44*), Israel (*45*), and France (*46*) since 2005. Possible reasons for underdetection and underreporting of HAdV-55 are its recent designation in 2013 as a discrete adenovirus type, following its recognition as an intertypic recombinant (P14H11F14) (*17*), and probably also molecular typing practices based solely on partial sequencing of the hexon gene. Molecular diagnosis of HAdV-55 and other intertypic recombinant HAdV genotypes requires a PCR-based assay targeting >2 regions of the genome, the penton base and hexon or the hexon and fiber genes; such assays would greatly improve molecular surveillance practices. The prototype strain, 273/Spain/1969, was detected in association with a severe ARD outbreak involving military recruits in Spain and reported as a serologically intermediate variant 11/14 (*34*). The US strain 97026382/South Dakota/1997, originally reported as HAdV-11 (*35*) and subsequently described as genome type 11a (*16*), caused a large ARD outbreak in a job training center. It is notable that the circulation of these viruses was not detected or reported in Europe or North America in the years following the detection of either the prototype or US strain. On the other hand, the long persistence of HAdV-55 in China and South Korea suggests continuous transmission and endemicity. If strains circulating in China or South Korea can cause repeated outbreaks, that indicates an important change in the epidemiologic pattern of HAdV-55 infection: from sporadic epidemic outbreak to persistent endemicity. The identification of factors that affect HAdV-55 transmission, from novel functional changes in the viruses to social or environmental changes, warrants further investigation.

Our study and others have shown that recent HAdV-55 genomes share a nucleotide identity >99.7% (*22*,*23*,*29*). The nucleotide variations are located all over the genome and are not concentrated in any particular genomic regions. We noted amino acid substitutions on proteins essential for viral replication, such as L4 100K, which may affect virus growth phenotype, antigenicity, infectivity, or virulence. Terminal protein precursor (pTP) and its protease-processed derivatives, intermediate terminal protein (iTP) and mature terminal protein (TP), play crucial and complex roles in adenovirus genome replication and virus maturation. The mutation P18S is unique to South Korea strains and located in a conserved region of pTP. Because the structure of pTP protein of adenovirus has not been resolved, it is unknown whether the P to S mutation will affect pTP structure and consequently change protease cleavage pattern of pTP, DNA replication, and genome packaging. Earlier work by Hay et al. (*47*,*48*) demonstrated that point mutations and deletions generated on pTP affect DNA replication activity in vitro. Flint et al. (*49*) showed that G315V substitution in pTP of HAdV-5 impaired pTP maturation leading to reduced infectivity. The remaining genomic changes of HAdV-55 appear to be largely trivial and not likely responsible for the increased incidence of outbreaks and disease severity. Further investigation and comparison of these strains on virology, molecular biology, and biochemistry perspectives will provide solid evidence to clarify whether some of the current strains are more infectious or virulent and therefore pose higher risks to human health. Detailed molecular epidemiology study, such as reported by Jing et al. on household HAdV-55 transmission (*50*), is warranted to enhance etiologic understanding of HAdV-55–caused ARD for accurate and timely diagnosis and disease prevention.

The United States has the highest number of domestic and international trade and travel visits in the world and is therefore highly susceptible to importation and dispersion of incoming pathogens such as HAdV-55. Indeed, isolated HAdV-55 cases and at least one outbreak have occurred in the United States with no clear identification of the source. Of interest, as suggested by the results of our phylogenetic analysis, the 2 US strains were not apparently related to each other. Therefore, implementation of enhanced surveillance, including typing of clinically relevant HAdV strains, is needed, along with proper design of countermeasures such as rapid diagnostics, treatments, and novel vaccines.

A limitation of this study is the lack of detailed clinical data and travel history. More information is needed for accurate risk assessment of disease transmission. It is unclear whether NHRC557006/Japan/2017 was introduced from South Korea by travel, or whether a cryptic circulation of the South Korea strain in Japan has yet to be detected. Similarly, without reports on additional HAdV-55 cases in Japan, it remains unknown which HAdV-55 strain, the South Korea strain, WPAFB415/Japan/2012, or other unknown strains, are circulating in Japan. A large number of HAdV-55-associated ARD cases were detected in both South Korea military personnel and US military personnel stationed in South Korea. Determining whether infected persons trained together or participated in same military events, how long and how often they were in close contact, the timeline of infection, and whether ARD outbreaks during an extended training period were underreported is important. It is worth noting that US active duty military in South Korea were vaccinated against HAdV-4 and HAdV-7, which suggests that the HAdV-4 and HAdV-7 vaccine formulation does not confer adequate protection against HAdV-55. Our ongoing and planned studies on HAdV-55–specific serologic surveys before, during, and after military deployment, as well as local serologic surveys in South Korea and Japan, will contribute to comprehensive understanding of HAdV-55 prevalence and enable data-driven decisions on the necessity of enhanced surveillance and development of effective prophylaxes.

Appendix Additional information for study of human adenovirus type 55 distribution, regional persistence, and genetic variability.
